# Functional MRI of visual cortex predicts training-induced recovery in stroke patients with homonymous visual field defects

**DOI:** 10.1016/j.nicl.2021.102703

**Published:** 2021-05-21

**Authors:** J.A. Elshout, D.P. Bergsma, A.V. van den Berg, K.V. Haak

**Affiliations:** a Donders Institute for Brain, Cognition and Behaviour, Radboud University Medical Centre, Nijmegen, The Netherlands

**Keywords:** Stroke, Visual field defects, Rehabilitation, Training, fMRI

## Abstract

Post-chiasmatic damage to the visual system leads to homonymous visual field defects (HVDs), which can severely interfere with daily life activities. Visual Restitution Training (VRT) can recover parts of the affected visual field in patients with chronic HVDs, but training outcome is variable. An untested hypothesis suggests that training potential may be largest in regions with ‘neural reserve’, where cortical responses to visual stimulation do not lead to visual awareness as assessed by Humphrey perimetry—a standard behavioural visual field test. Here, we tested this hypothesis in a sample of twenty-seven hemianopic stroke patients, who participated in an assiduous 80-hour VRT program. For each patient, we collected Humphrey perimetry and wide-field fMRI-based retinotopic mapping data prior to training. In addition, we used Goal Attainment Scaling to assess whether personal activities in daily living improved. After training, we assessed with a second Humphrey perimetry measurement whether the visual field was improved and evaluated which personal goals were attained. Confirming the hypothesis**,** we found significantly larger improvements of visual sensitivity at field locations with neural reserve. These visual field improvements implicated both regions in primary visual cortex and higher order visual areas. In addition, improvement in daily life activities correlated with the extent of visual field enlargement. Our findings are an important step toward understanding the mechanisms of visual restitution as well as predicting training efficacy in stroke patients with chronic hemianopia.

## Highlights

• Damage to the visual brain typically leads to vision loss.
• Vision loss may be partially recovered with visual restitution training (VRT)
• Cortical responses to visual stimulation do not always lead to visual awareness.
• A mismatch between Humphrey and neural perimetry predicts training outcome.
• This finding has important implications for better rehabilitation strategies.

## 1 Introduction

Post-chiasmatic damage to the visual system leads to homonymous visual field defects (HVFDs), which can range from small scotomas to a loss of visual functioning in the entire contralateral hemifield (hemianopia). HVFDs can severely interfere with daily life activities, such as reading, driving, object avoidance and usage of computers (Gall et al., 2010, Papageorgiou et al., 2007, Zihl, 2010), thereby leading to poorer social interactions, mobility and job security. In the first three months after brain injury spontaneous recovery may occur (Zihl, 2010, Zhang et al., 2006), after which assiduous visual restitution training (VRT) may further improve visual functioning (Kasten et al., 1998, Huxlin et al., 2009, Marshall et al., 2010, Bergsma and van der Wildt, 2010, Das and Huxlin, 2010, Plow et al., 2012, Elshout et al., 2016). The extent of improvement, however, varies greatly among patients, and it is so far unclear what factors determine these inter-individual differences. Identifying these factors is an important step toward understanding the mechanisms of visual recovery after stroke, and to leverage these insights to improve visual rehabilitation.

The amount of visual field loss, and its change over time or after training, is typically assessed using automated perimetry. It is a standard visual field test in clinical ophthalmology that reveals at what locations in the visual field the patient has visual awareness by showing flashes of light of varying intensities while the subject presses a button when they see the light. Thus, it is a subjective behavioral measure that summarizes visual functioning along the entire visuomotor system. An alternative way of performing a visual field test is by recording the neural responses to visual stimuli presented at different visual field locations. Just as the button presses during automated perimetry can be converted to maps of visual field locations that indicate the subject’s visual sensitivity, the neural responses can be converted to maps of visual field locations at which different stages of visual processing are responsive to visual stimulation. In contrast to standard behavioral perimetry, neural perimetry (by retinotopy mapping) offers an objective characterization of visual sensitivity. Population receptive field mapping is a widely accepted approach in ophthalmic science to study changes in visual cortical responses after visual field loss in a variety of patient groups (Miranda, et al., 2018, Silson et al., 2018, Carvalho et al., 2021, Sims et al., October 2020, Halbertsma et al., 2019, Haak et al., 2014, Haak et al., 2012, Baseler et al., 2011).

Aside from methodological differences, an important distinction between behavioral and neural perimetry is that they characterize visual sensitivity at different stages of visuomotor processing. Thus, the ensuing maps may also be different. For instance, as demonstrated in recent work (Papanikolaou et al., 2014), a scotoma in terms of behavioral perimetry may not be visible in perimetric maps based on primary visual cortex (V1) responses in patients with higher order visual cortex lesions. In similar vein, a case was presented where focal injury to V1 perturbed visual perception, even though the neural measurements from that area indicated that it still responded to visual stimulation (Papanikolaou et al., 2014). In this case, it appeared that the extant neural responses came from spared islands of functional tissue that were too weak or disorganized to elicit a visual percept during behavioral perimetry. It is further possible that the neural perimetry indicates an absence of visual processing at locations that do elicit a behavioral response. This could be due to the use of locally weaker visual contrast during neural than behavioral perimetry, measurement noise, neural signaling pathways bypassing the neural recording site, or noisy neuronal signals that are ‘denoised’ at later visual processing stages (e.g. by spatial pooling of many noisy signals).

An important -yet untested- hypothesis is, that the visual field locations at which the behavioral perimetry indicates vision loss while the neural perimetry does not point to a ‘neural reserve’, offering greater potential for visual rehabilitation. Here, we tested this hypothesis in stroke patients with chronic hemianopia who followed an intensive VRT program. Importantly, we elected to perform neural perimetry based on functional MRI measurements across the entire visual hierarchy to avoid otherwise inevitable issues with visual area identification in stroke patients with occipital lesions, and to deal with the fact that each patient is different in terms of lesion location and size. Furthermore, unlike most functional MRI investigations into the human visual system, we adopted a wide-field stimulus presentation approach that allowed us to perform neural perimetry across at least the same extent of the visual field as standard visual field testing in the clinic. Our results indicate that VRT leads to strong and replicable training effects at field locations with neural reserve, while training effects were small and statistically insignificant across the rest of the visual field. Thus, a relatively inexpensive and minimally demanding functional MRI scan can provide important complementary information to standard visual field testing allowing for a more efficient, personalized VRT program as well as improved assessments of a patient’s potential for rehabilitation.

## 2 Methods

### 2.1 Participants

Forty patients (seven female) with HVDs as a result of stroke were included in this study (ages between 26 and 75). All patients were in the chronic phase of stroke (>10 months post stroke), to exclude spontaneous recovery (Zhang et al., 2006), and they had no other visual anomalies that could not be corrected for (e.g. macular degeneration, glaucoma, cataract) or unilateral spatial neglect as assessed by the line bisection test. They all gave written informed consent. The study was conducted in accordance with the Declaration of Helsinki and approved by the local ethics committee CMO Arnhem-Nijmegen.

### 2.2 Intervention

**Fig. 1:**
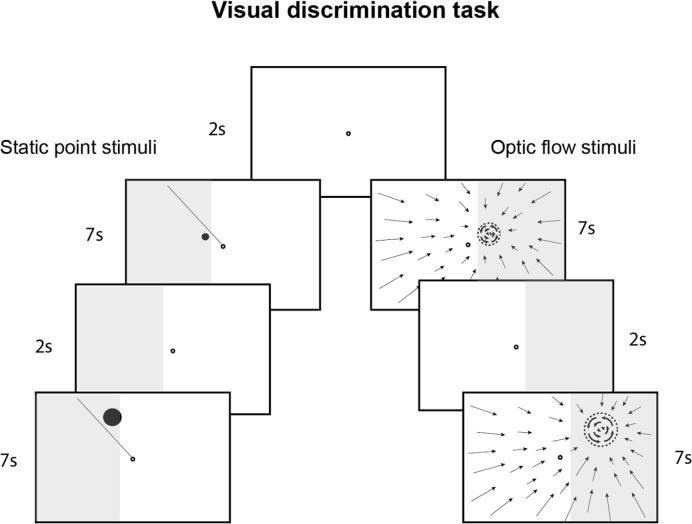
Visual discrimination task. Patients trained either with static point stimuli or optic flow stimuli (moving dots that were presented continuously on screen). The stimulus was presented on the border area of the visual field defect (gray shaded area) and scaled with eccentricity. Patients reported whether the stimulus location was clockwise or counterclockwise located with respect to the line extending from the fixation target (static point stimulus) or rotated clockwise/counterclockwise (optic flow pattern). The target location was cued by the line or background contraction pattern in order to make a directed covert attention shift.

Each patient received 80 h of a 2-AFC visual discrimination training. Detailed description of the training can be found in Elshout et al. (2016) (Elshout et al., 2016). In short, the patient maintains fixation on a central fixation ring (diameter = 0.5 deg) and makes a covert attention shift to a stimulus presented on the border area of the visual field defect. The stimulus was either a static dot (0.2 deg at 1 deg eccentricity) that was presented clockwise or counter clockwise relative to a line extending from the fixation ring (difference of 10 deg meridional angle), or an optic flow pattern (1.7 deg at 1 deg eccentricity) rotating clockwise or counter clockwise about the training location (Fig. 1). The optic flow stimulus was generated using openGL (van den Berg, 1996). A 3D volume (width × height × depth: 4 × 4x 1) was filled with random dots that were moving at constant speed parallel to the simulated heading direction. When a dot left the volume it was replaced at a random location in the opposite border plane. This gives rise to a continuous flow with a radial structure centered on the simulated heading direction. For the target stimulus, an independent volume of dots was moved likewise with a dominant (CW/CCW) rotational flow component added. The two sets of dots were projected on the screen through complementary masks. The dots were in either simulation of constant size. The target dots were always smaller in proportion to the size of the target patch, which depended on eccentricity.

Importantly, the stimulus location within the affected hemifield was cued by the direction of the line extending from the fixation target or the origin of a contracted optic flow stimulus (group of moving dots) presented continuously on the entire screen. Both stimuli (white on a black screen) were presented for 7 s and scaled with eccentricity following the cortical magnification factor (Cowey and Rolls, 1974). The patient responded ‘clockwise’ or ‘counter clockwise’ using the arrow keys on a keyboard. Fixation was monitored using a webcam and software available in the public domain (http://www.inference.org.uk/opengazer). A trial where fixation was lost was repeated at the end of each session (~12 min, between 60 and 100 stimuli). The patients trained 1 h a day, 5 days a week during 16 weeks. Thirty patients received 40 h of training in the affected hemifield and 40 h of training in the intact hemifield. The other 10 patients received 80 h of training in their affected hemifield (field defect split in half and trained for 40 h each). Before and after training, Humphrey perimetry and wide field retinotopic mapping and Goal Attainment Scaling (GAS) was conducted.

### 2.3 Humphrey perimetry

Before and after training we performed visual field testing using Humphrey perimetry. To enable blind spot probing (which is necessary for assessing the reliability of the results), this was done for the eye where the blind spot did not fall within the patient’s scotoma. We applied the SITA fast 30–2 program of the Humphrey Field Analyzer II in a standardized way to collect sensitivity maps for the central 30° of vision. This program probes 76 locations (38 per hemifield), starting from 3° from to fovea with steps of 6° onto 27° in the periphery. Humphrey perimetry reports remaining visibility on a logarithmic scale ranging from 0 dB to a subject specific maximum visibility of about 35 dB. Improvement after training was specified in terms of the average dB gain across all locations and in the number of locations that improved by at least 1 dB.

### 2.4 Neural perimetry

**Fig. 2:**
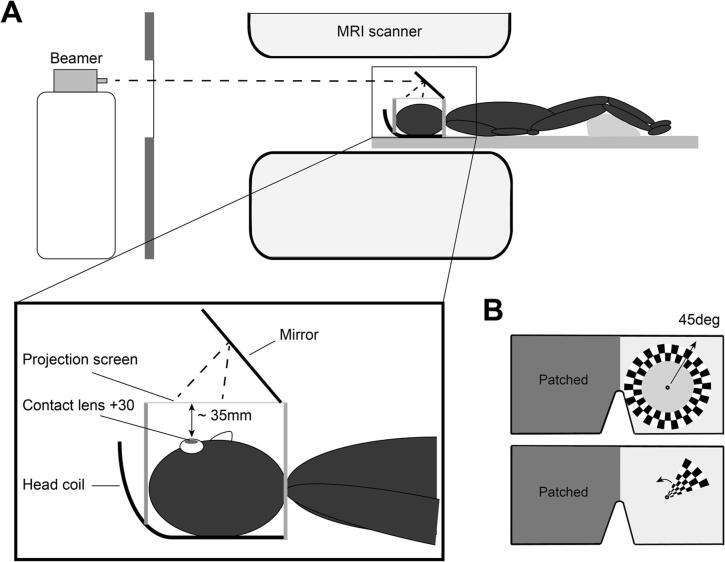
Scanning paradigm. (A) Setup for wide-field retinotopic mapping inside MRI scanner. (B) Retinotopic mapping stimulus presented inside the MRI scanner. The ring stimulus extended from the fixation target (centered in front of the right eye position) to 45 deg of eccentricity. The wedge stimulus rotated counterclockwise around the fixation target.

A detailed description of the wide field retinotopic mapping procedure and pRF analysis can be found in Elshout et al. (2018) (Elshout et al., 2018). In short, we presented visual stimuli on a custom built projection screen placed about 3 cm above the patient’s eye (field of view ~ 90°x90°). The patient wore a custom made soft convex lens with a refractive power of + 30 diopters in the eye opposite to the affected hemifield (same eye as used in Humphrey perimetry) to allow effortless sharp vision at the screen (Fig. 2A). While maintaining fixation on a central fixation ring, a full contrast, ‘‘wedge-shaped’’ checkerboard stimulus (maximum angular width of ~ 45°) rotated counterclockwise about the center of fixation at one cycle in 64 s (Fig. 2B). For the eccentricity mapping, a radial full contrast checkerboard ring stimulus moved from the center towards the periphery of the visual field at one revolution in 64 s (maximum eccentricity of ~ 45 deg). For both mapping stimuli, contrast was reversed at a frequency of 2 Hz. The checks and the width of the ring-stimulus were scaled by eccentricity in accordance with the cortical magnification factor (Cowey and Rolls, 1974). The patient maintained fixation on a central fixation ring during stimulation. We collected at least 3 runs of ~ 4.5 min for each stimulus for each patient. All data was collected on a 3 T Siemens TRIO or SKYRA system at the Donders Centre for Cognitive Neuroimaging (Nijmegen, The Netherlands). To obtain a high resolution full-brain anatomical scan (T1-weighted MPRAGE, 192 slices, 256 × 256 matrix, 1 × 1 × 1 mm resolution), we used the 32-channel head coil. During the experimental scan sessions, the occipital part of the head coil with 20-channels was used to enable the wide-field screen presentation. High-resolution functional scans were obtained with an in-plane resolution of 2 mm *iso*-voxel and a slice thickness of 2 mm (T2*-weighted; multi-echo echo planar imaging; 32 slices; repetition time of 2 s; echo time of 28 ms). All experimental scan sessions were collected on the same day for each patient (~1.5-hour scan session). The anatomical scan was collected on a separate day. In the last group of patients, we used an Eyelink 1000 (SR Research, Ltd.) to monitor fixation stability.

Following pre-processing of the MRI data (automatic segmentation, rendered as a smoothed 3D surface and motion correction between and within functional scans), the data were loaded into the MRVISTA toolbox environment (http://white.stanford.edu/software) and the functional data were aligned with the whole-brain anatomical segmentation. Next, the time-series data were averaged across scans, separately for each stimulus, resulting in two average-time series per subject (i.e. one for the wedge stimulus, and one for the ring stimulus), which were subsequently resampled to match the 1 mm isotropic resolution of the gray/white matter segmentation using trilinear interpolation. Population receptive field (pRF) parameters were estimated according to procedures described by Dumoulin and Wandell (Dumoulin and Wandell, 2008). For each voxel, fMRI time-series predictions were generated by varying a wide range of plausible values for the parameters (x, y, σ) of a circularly symmetric Gaussian pRF model. Optimal parameters were identified as those that minimized the residual sum of squares between the time-series data and prediction. The best-fitting pRF model parameters were converted to polar coordinates expressed in visual angle. Finally, we converted the pRF model estimates to neural perimetry maps using all voxels whose best-fitting pRF model explained at least 20% of the variance in the time-series.

### 2.5 Comparison of Humphrey and neural perimetry maps

**Fig. 3:**
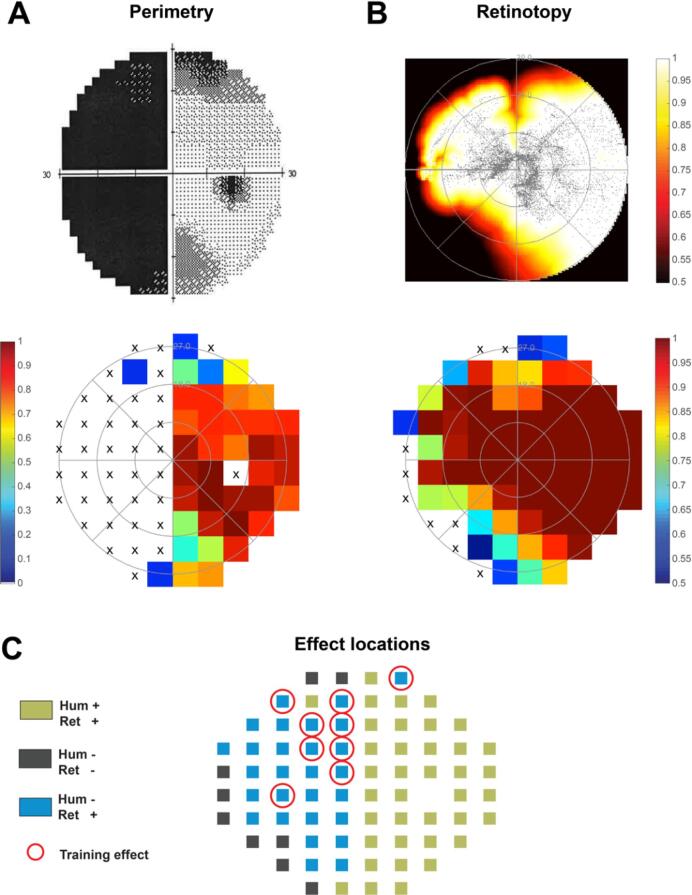
(A) Comparison of Humphrey and neural perimetric coverage maps. This subject (J01) has been diagnosed with a complete left sided hemianopia based on Humphrey perimetry. The lower panel is normalized to this subject’s maximum visibility. An ‘x’ marks the Humphrey test locations with no response (i.e. 0 dB). (B) However, based on the patients’ coverage map resulting from retinotopic mapping the hemianopia is partial with a largely spared upper left quadrant. Following to the hypothesis of Papanikolaou et al. this upper quadrant may be susceptible for functional recovery. The lower panel is down sampled from the upper panel to match the resolution of the Humphrey map and thresholded at 0.5. An ‘x’ marks the Humphrey grid locations with a maximum amplitude of the Gaussian pRF across all voxels inside this grid location below threshold (i.e. 0.5). (C) Four categories were classified based on the lower two panels in A and B: (1) Hum + / Ret + represent coverage for both the retinotopy and Humphrey (coloured grid location in both maps); (2) Hum - / Ret - represent no coverage in either map (‘x’); (3) Hum - / Ret + represent coverage for the retinotopy map only (coloured grid in retinotopy; ‘x’ in Humphrey); (4) Hum + / Ret - represent coverage for the Humphrey map only (coloured grid in Humphrey; ‘x’ in retinotopy. Because this last category is infrequent (1.5% of the data), we excluded this category from further analysis. The three categories are shown for the patient used in this example (J01). The red circles indicate the locations with a training effect (i.e. 0 dB prior to training and > 1 dB after training). Note, the blind spot location is excluded. (For interpretation of the references to colour in this figure legend, the reader is referred to the web version of this article.)

First, all neural perimetry maps were down-sampled to the resolution that matches the Humphrey perimetry maps. The Humphrey test locations lie on a 10x10 grid with 6° spacing. Therefore, we

determined the maximum amplitude of the Gaussian pRF (normalized between 0 and 1) across voxels at each visual field location (6° x 6° pixel) that matched a Humphrey test location, thus creating a single neural perimetry map for each patient. The resulting retinotopic map were thresholded at 0.5 (Fig. 3). Next we classified each location (*N* = 76) in one of four categories: (a) there was coverage (neural map > 0.5, Humphrey map > 0 dB) for the retinotopic map and Humphrey map (Hum + / Ret + ), (b) coverage for Humphrey but not retinotopy (Hum + / Ret -), (c) coverage for retinotopy but not Humphrey (Hum - / Ret + ) or (d) no coverage in both maps (Hum - / Ret -). Last, we calculated the effect of training by subtracting the Humphrey measurement after training from the Humphrey before training. To find out how the overlap category affected the success of the training, the average dB visual field improvement per grid location was calculated for each overlap category per subject and averaged across patients.

### 2.6 Goal Attainment scaling

In order to relate perimetry results with personal improvement in daily life activities, GAS was applied by three independent occupational therapists, who were blinded to training outcome. A more detailed description about how GAS was applied can be found in Elshout et al (2018) (Elshout et al., 2018). In brief, each patient sets three SMART (Specific, Measurable, Achievable, Realistic, Time orientated) goals prior to training under supervision of the therapist (Turner-Stokes and Ashford, 2007). After training these goals were evaluated together with the therapists using a 6-point Likert scale. The achievement after training could be worse (–3), the same (–2), somewhat better but goal not achieved (–1), goal achieved (0), better than goal (+1) or much better than goal (+2). The relation between GAS outcome score and visual field improvement was assessed using linear regression.

### 2.7 Statistical analyses

Paired sampled t-tests were used to compare Humphrey test characteristics before and after training. A permutation test with 10,000 iterations was used to compare the training effects between the different categories. Specifically, during each iteration, we randomly permuted the category labels (Hum - / Ret -, Hum - / Ret +, Hum + / Ret -, Hum + / Ret + ) associated with each visual field location and recomputed training effect (second Humphrey measurement minus first Humphrey measurement) for the permuted categories to determine the null distribution of the training effect differences. The p-value was then determined by comparing the training effect for the unpermuted categorization against the established null distribution. A regression analysis was used to estimate the linear relationship between GAS score and training effects. A p-value < 0.05 was considered as statistically significant.

## 3 Results

Six patients dropped out during training and seven were excluded from further analysis due to unreliable perimetry based on the standard clinical criterion (i.e. default setting of the Humphrey perimeter) of > 20% false detection during blind spot probing (Khan et al., 2011). Thus, 27 patients were included in the analysis (mean age 51.8 years, seven females). The study followed a randomized controlled crossover design (Elshout et al., 2016), wherein each patient engaged in two training rounds during which they trained either inside and outside, or at two complementary halves of the perimetric scotoma (see Table 1 for details). The number of training locations was always equal for the two training rounds. Stimulus type and training region order were randomized across patients and established prior to the inclusion of the first patient.

**Table 1:** Patient Information.

Subject	Gender	Age (years)	Time since lesion (months)	Field defect	Lesion	Cause (stroke)	Training paradigm a, b
Group A							
J01	M	66	27	Hemi-L	R occipital/parietal cortex	Ischemic	Intact
J02	M	61	12	Scot-L	R occipital cortex	Ischemic	Defect
J03	M	61	34	Hemi-R	L occipital cortex	Ischemic	Intact
J05	M	56	18	Quadr-lower-R	L occipital/parietal cortex	Ischemic	Defect
J06	M	69	17	Hemi-R Inc.	L occipital cortex	Ischemic	Defect
J07	F	43	20	Hemi-R Inc.	L occipital cortex	Ischemic	Intact
J08	F	57	17	Hemi-R	L occipital cortex	Ischemic	Defect
J41^c^	F	46	22	Hemi-R Inc.	L occipital cortex	Ischemic	Intact
J12	M	52	29	Hemi-L Inc.	R occipital cortex	Ischemic	Defect
J14	M	54	16	Quadr-lower-R	L occipital cortex	Ischemic	Intact
J15	M	33	18	Hemi-R	L temporal cortex / optic rad.	Hemorrhagic	Defect
J16	F	43	21	Hemi-R Inc.	L occipital cortex	Ischemic	Intact
J17	M	43	19	Hemi-LR Inc.	R + L occipital cortex	Ischemic	Intact
J18	M	48	17	Scot-upper-L	R occipital cortex	Ischemic	Defect
J20	M	53	17	Hemi-L Inc.	R temporal / parietal cortex (optic rad.)	Ischemic	Defect
J24	F	46	111	Hemi-L Inc.	R occipital cortex	Ischemic	Intact
J25	M	48	19	Quadr-upper-L	R occipital cortex	Ischemic	Intact
J26	M	29	21	Hemi-R Inc.	L occipital cortex	Ischemic	Defect
J27	M	49	38	Hemi-R	L occipital/temporal cortex	Hemorrhagic	Defect
J28	M	68	19	Hemi-L Inc.	R occipital cortex	Ischemic	Intact
J30	M	34	11	Hemi-L Inc.	R occipital/parietal cortex	Hemorrhagic	Intact
Group B							
J31	F	57	45	Hemi-R Inc.	L occipital cortex	Hemorrhagic	Upper
J32	M	44	32	Hemi-L Inc.	R occipital/temporal cortex	Hemorrhagic	Upper
J33	M	75	30	Scot-R	L occipital cortex	Ischemic	Lower
J35	M	68	20	Scot-L	R occipital cortex	Ischemic	Upper
J38	F	47	27	Hemi-R Inc.	L occipital cortex	Ischemic	Upper
J40	M	26	16	Hemi-L	R occipital/temporal cortex	Hemorrhagic	Lower

Abbreviations: Hemi, hemianopia; Quadr, quadrantanopia; Scot, scotoma; L, left; R, right; Inc, incomplete.

a Intact = intact training first; Defect = defect training first

b Upper = upper quadrant training first; Lower = lower quadrant training first

c J11 dropped out the study and was replaced by J41

### 3.1 VRT improves visual sensitivity in chronic hemianopia

Patients trained at home using a custom built training unit for approximately one hour per day, five days a week, completing eighty hours of training after sixteen weeks of training in total (eight weeks per training type). They performed a 2-alternative forced choice visual discrimination task at many different locations within the targeted visual field region (60–100 trials per ~ 12 min session depending on the size of the target region) while maintaining fixation on small (.5deg) ring at the center of the screen. Stimuli were shown for 7 s, during which the patients covertly shifted their attention toward the stimulus location (i.e. while maintaining fixation). Prior to the start of the study, patients were randomly assigned to train with either a static point stimulus, or an optic flow discontinuity stimulus during both training rounds. The static point stimulus consisted of a white point (of at least .2deg and size scaled with eccentricity) presented on a black background. Patients indicated whether the point stimulus appeared clockwise or counterclockwise with respect to a line extending from the fixation point, which served as a cue for the location of the point stimulus. The optic flow stimulus consisted of a small circular patch of rotating dots (at least 1.7 deg and scaled with eccentricity) embedded in an optic flow pattern that covered the entire visual field contracting into the patch location. Patients indicated whether the dots at the patch location rotated clockwise or counterclockwise. Because our hypothesis is not stimulus specific and there were no systematic differences between the training effects associated with the two training stimuli (Elshout et al., 2016), we pooled the data across the two training stimuli to increase statistical power.

Humphrey automated perimetry maps were obtained before and after training according to the SITA-fast 30–2 protocol using research staff blinded to the experimental conditions. The perimetry was performed monocularly to the eye opposite to the affected visual hemifield. Though manual Goldmann perimetric maps were also obtained for most patients, we elected to characterize the training effects using automated Humphrey perimetry, because automated perimetry is less prone to possible experimenter bias and hence the current standard in clinical assessments and research trials.

Overall, the quality of the Humphrey perimetry for the patients that were included in the study was excellent, with very few fixation errors, false positives or negatives, and no significant differences between sessions (all t(26) < 1.108, p > 0.27). See Supplementary Table S1 for details. Supplementary Figure S1 shows the resulting perimetry maps for each individual. Training effects were established for each individual and each grid location by computing the dB change in visual sensitivity. On average, across all 27 subjects and across all perimetric grid locations, training significantly improved visual sensitivity by 0.90 ± 0.10 dB. A test–retest reliability analysis to establish the stability of the perimetric maps over at least 28 days in an independent group of six stroke patients (mean age 53.2 years) with chronic hemianopia who received no training at all showed a change of 0.11 ± 0.16 dB which is significantly lower than the changes observed under training (p < 0.001). Thus, even when including intact visual field locations where little improvement might be expected, VRT significantly increases average visual sensitivity in chronic hemianopia.

### 3.2 VRT is most effective at locations in the perimetric scotoma that still drive visual cortex

The perimetry map obtained prior to training was compared against a visual field coverage map based on a wide-field retinotopic mapping functional MRI experiment as detailed in (Elshout et al., 2018). During the wide-field retinotopic mapping experiment, the patients stared at high-contrast expanding ring and rotating wedge checkerboard stimuli. To achieve wide-field coverage of at least 30°eccentricity, we used a custom built visual projection system that allowed us to present the stimulus images at about 3 cm above the patient’s eyes. The patients viewed the stimuli monocularly as they wore a + 30 diopter lens in one eye to enable effortless, sharp nearby vision. To create visual field coverage maps that can be compared against the perimetry maps, we performed standard population receptive field (pRF) mapping procedures, characterizing functional MRI responses as a function of visual stimulus location. Notably, these retinotopy-based visual field coverage maps were not limited to specific visual areas, because delineating these areas is challenging in the face of brain lesions and not necessary to test the hypothesis. The retinotopy maps were down-sampled to match the resolution of the perimetry maps by averaging values of voxels covering a particular (6° x 6°) grid location irrespective of their cortical location. Both maps were then normalized to range between 0 and 1 by dividing them by the maximum value across all visual field locations (Fig. 3). Next we classified each visual field location (*N* = 76) in one of four categories: (Hum + / Ret + ) coverage for both the retinotopy (>0.5) and Humphrey map (>0 dB); (Hum + / Ret -) coverage for the Humphrey map only; (Hum - / Ret + ) coverage for the retinotopy map only; (Hum - / Ret -) no coverage in either map. An example is shown in Fig. 3C. The effect of training was calculated by subtracting the Humphrey map after training from the Humphrey map before training.

**Fig. 4:**
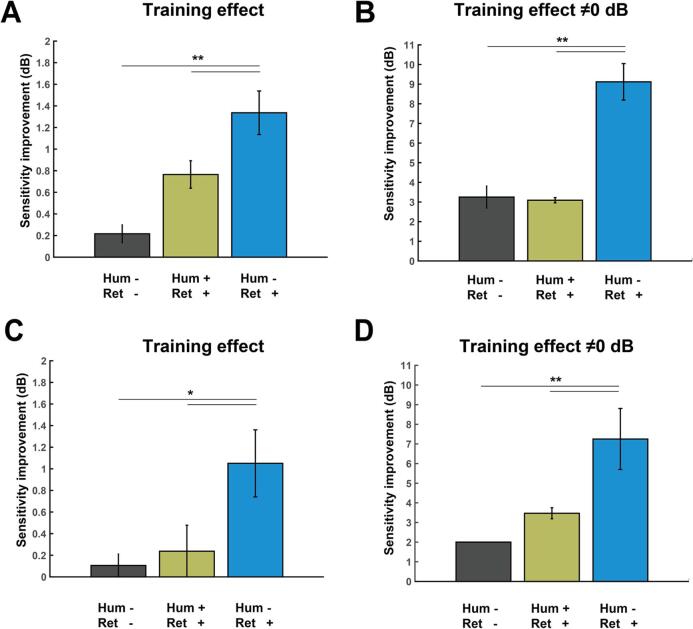
Training effects at different visual field locations. (A) Training effects established by Humphrey perimetry (map after training – map prior to training) averaged across all locations from the corresponding category from all patients. (B) Training effects established by Humphrey perimetry averaged across all locations from the corresponding category from all patients that show at least 1 dB visual sensitivity improvement; i.e. locations with exactly the same dB value after training (change = 0 dB) excluded. (C) Training for patients of cohort 4 who received training in the complementary halves of the scotoma (did not received training in the intact field). (D) Training effects for patients of cohort 4 established by Humphrey perimetry averaged across all locations from the corresponding category from all patients that show at least 1 dB visual sensitivity improvement; i.e. locations with exactly the same dB value after training (change = 0 dB) excluded. Note: only positive fluctuations for the green category (Hum+/Ret + ) are shown, since the other categories are also clipped at 0. (For interpretation of the references to colour in this figure legend, the reader is referred to the web version of this article.)

We first considered the perimetric and neural data of the 21 patients who trained inside as well as outside their perimetric scotomas. The Humphrey perimetry and retinotopy maps agreed on the state of the visual field (Hum + / Ret + or Hum - / Ret -) at 69.4% of all tested visual field locations across all patients. In 29.1% of all tested locations the Humphrey outcome showed no response while retinotopy did show coverage (Hum - / Ret + ). In 1.5% of all tested locations, Humphrey outcome showed a location > 0 dB while retinotopy indicated no coverage. Because this last category is infrequent and difficult to interpret (it might involve noisy pRF estimates), we excluded this category from further analysis. For each of the remaining categories, we calculated the mean Humphrey dB change after training across all locations and patients. This analysis revealed that the largest visual field improvement by training was found in the loci classified as Hum - / Ret +, i.e. visual field locations with neural reserve (Fig. 4A). A permutation test with 10,000 iterations showed that the training effect at locations with neural reserve (1.34 ± 0.20 dB) was significantly larger than at locations classified as Hum + / Ret + (0.76 ± 0.13 dB) and Hum - / Ret - (0.22 ± 0.08 dB) (both p < 0.01). Repeating this analysis whilst including only test locations that changed after training (i.e. dB change of 0 dB excluded, Fig. 4B) led to the same conclusion with a strong training effect that was significantly larger at the Hum - / Ret + locations (9.12 ± 0.93 dB) than for the other two categories (Hum + / Ret + = 3.09 ± 0.13 dB and Hum - / Ret - = 3.25 ± 0.56; both p < 0.005).

These results could be replicated in the six patients who trained at two complementary halves of their perimetric scotoma as well as for different neural detection thresholds. For the second group of patients, the Humphrey perimetry and retinotopy maps agreed on the state of the visual field (Hum + / Ret + or Hum - / Ret -) at 69.7% of all tested visual field locations across all patients, and in 30.3% of all tested locations the Humphrey outcome showed no response while retinotopy did show coverage (Hum - / Ret + ). For none of the tested locations Humphrey indicated sensitivity > 0 dB while retinotopy indicated no coverage. As before, also, the largest visual field improvement by training was found in the loci classified as Hum - / Ret +, because the training effect at these visual field locations (1.05 ± 0.31 dB) was significantly larger (p = 0.026) than at locations classified as Hum + / Ret + (0.24 ± 0.24 dB) and Hum - / Ret - (0.11 ± 0.11 dB). Including only test locations that changed after training (i.e. dB change of 0 dB excluded, Fig. 4C and D) again led to the same conclusion with a strong training effect at the locations with neural reserve (Hum - / Ret + = 7.25 ± 1.55 dB) that was significantly larger (p < 0.005) than the gains for the loci of the other two categories (Hum + / Ret + = 3.47 ± 0.28 dB and Hum - / Ret - = 2 dB (this last category encloses only one data point). Importantly, while many locations on the border area of the Ret+/Hum- location improve, there are also many locations deeper in the scotoma that improve (e.g. J01, J17, J31), which show that it is not an artefact on the border area exclusively. In addition, and most crucially, the border area of the scotoma is also represented in the Ret-/Hum- condition, so it is unlikely that artefacts on the border area explain the difference between these two conditions. Supplementary Fig. S2 further shows that the increased training effects at neural reserve locations can also be observed when the neural signal detection threshold was set to 0.25 or 0.75 instead of 0.5. Thus, the mismatch between Humphrey and neural perimetry prior to training robustly identifies visual field locations that can be recovered by training.

### 3.3 Idiosyncratic neural underpinnings of the VRT effects

Due to the technical challenges associated with accurate delineation of specific visual areas in patients with occipital lesions, the retinotopy-based visual field coverage maps were not limited to specific visual areas such as V1. To still gain insight into whether the observed training effects might be underpinned by specific visual areas, we mapped the dB change after training back onto the cortical surface of each individual. Importantly, this was done based on the—possibly non-unique—mapping between cortical and visual field locations provided by the pRF estimates. That is, each visual field location can in principle drive multiple cortical sites, so the cortical projection maps should be interpreted accordingly: they show the dB change at all cortical locations that responded to visual stimulation at a particular visual field location. Therefore, these maps ought to be interpreted as distinguishing between cortical regions that could and could not have supported the training effects, rather than precisely localizing the neural correlates of the training effects.

**Fig. 5:**
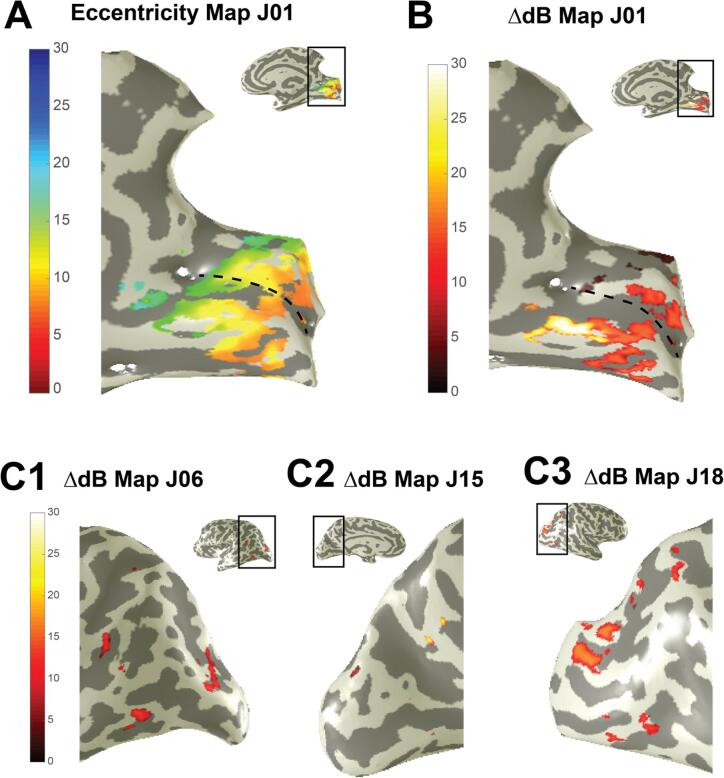
As can be deducted from panel (A) and (B), the locations with a training effect of patient J01 (on average about 12 dB) were mainly induced by regions in the primary visual cortex corresponding to peripheral visual information processing (dashed line indicate the calcarine sulcus). (C) However, training effects could also be induced by areas higher up in the visual processing stream (C1-3) in other patients.

Fig. 5A and B show as an example the cortical projections of the perimetric dB change for patient J01. In this patient, the projections highlighted primarily the early visual areas (Fig. 5B), which would be consistent with previous work suggesting that spared V1 regions may be susceptible for rehabilitation (Papanikolaou et al., 2014). However, inspection of all other patients showed more diverse projection patterns (e.g. Fig. 5, C1-C3 and Fig S3), implicating for instance regions on the dorsolateral and ventrolateral occipital surface. These projections are consistent with the idea that visual information can bypass the early visual areas, with shortcuts from LGN to higher order visual areas (e.g. V5) leading to some residual vision (Weiskrantz et al., 1974, Sahraie et al., 2006, Silvanto et al., 2007, Cowey, 2010). Thus, it appears that the neural underpinnings of the training effects cannot be always be attributed to a single visual area such as V1 or V5.

### 3.4 Association with goal-attainment scaling

In order to relate the VRT effects with personal improvement in daily life activities, goal attainment scaling (GAS) was performed for each patient by one of three independent occupational therapists, who were blinded to training outcome. GAS is a widely applied and oftentimes decisive measure of a potential treatment’s clinical significance. Each patient sets three SMART (Specific, Measurable, Achievable, Realistic, Time orientated) goals prior to training under supervision of the therapist (Turner-Stokes and Ashford, 2007). After training these goals were evaluated by the same therapist using a 6-point Likert scale. The achievement after training could be worse (–3), the same (–2), somewhat better but goal not achieved (–1), goal achieved (0), better than goal (+1) or much better than goal (+2). Previous work that assessed the relationship between the GAS scores and the average visual sensitivity change in dB across the affected visual hemifield did not establish a significant relationship (Elshout et al., 2018). Therefore, based on the present findings, we here focused on a subject’s neural reserve (i.e. all Hum-/Ret + locations). Linear regression analysis indicated no relation between GAS and the total amount of dB gained (*r* = 0.3, p = 0.145). However, we did establish a significant relation between the number of locations with neural reserve that improved, and the improvement on the GAS (*r* = 0.45, p = 0.023). These results underscore the clinical significance to patients with chronic hemianopia.

## 4 Discussion

We confirmed the hypothesis that visual field locations with neural reserve predict the loci of training-induced recovery in patients with homonymous visual field deficits due to stroke. Specifically, training effects were reproducibly larger at those visual field locations where standard behavioral perimetry indicated a visual field defect but fMRI did not. This result is important, because it indicates that minimally demanding fMRI scans can provide crucial information enabling a more focused visual training protocol. Indeed, it allows one to estimate how much of the perimetric scotoma might be recovered in a given patient, and where in the visual field that patient should be training to achieve maximal recovery with maximal efficiency. In addition to these clinical implications, the results shed light on the neural mechanisms by which vision can be partially restored by training, implicating patient-specific neural substrates at various cortical locations, which in turn suggests a fundamental capacity for neural plasticity across the human occipital lobe.

### 4.1 Clinical relevance

Few doctors currently advise their patients to embark on a long-term training schedule with uncertain outcome (ranging from no effect to partial recovery). Hence, the central motivation for the present work was to determine whether it be possible to identify patients with high expected training yield. By establishing that training yield is increased at locations with neural reserve, we established a possible imaging marker for identifying patients that are most likely to benefit from VRT and the field locations that should be targeted for training. Importantly, the observed training effects are not just statistically significant but also clinically meaningful. Indeed, the ‘trainable’ visual field locations gained about 9 dB in visual sensitivity on average far exceeded the intrinsic longitudinal variability of Humphrey perimetry in this type of patient, while the global increase across the entire visual field (+1dB) is of a similar magnitude to annual visual sensitivity changes seen in moderately progressing glaucoma (-0.5 to −1.5 dB) (Chauhan et al., 2008). Importantly, also, we established that the number of successfully trained visual field locations is predictive of a patient’s GAS score, which is a common measure in rehabilitation medicine to determine whether therapeutic treatments can actually meaningfully improve a patient’s functional outcome in daily life (Elshout et al., 2018, Turner-Stokes and Ashford, 2007, Bergsma et al., 2014).

Notwithstanding its clinical relevance, we note that the presence of neural reserve does not guarantee functional recovery: in five patients with dissimilar perimetric maps we found no training effect at all, and in patients that did exhibit training effects not all mismatch locations showed improvement. Understanding what prevented recovery in these cases is an important direction for future work. One possible explanation is task compliance. While we can confirm that all patients carefully followed the prescribed training program, we cannot rule out that some patients were distracted during the training sessions. It is also possible that some patients and/or visual field locations required more training than others. Analysis what determines the difference in restitution speed between patients and/or between training locations could improve the prediction potential of our technique. Here, it could be considered that differences in restitution speed might depend on patient-specific lesion locations. Also, the absence of training effects in specific patients could be related to an inability to learn the discrimination task or an inability to generalize improved performance during the discrimination task to improved stimulus detection during Humphrey perimetry.

Beyond the direct relevance to patients with chronic hemianopia, it is also worth mentioning that the present findings could be translated into a possible enrichment marker for clinical trials that aim to test the efficacy of particular VRT approaches, as well as for fundamental research studies aimed at understanding the neural mechanisms underlying visual field recovery by training so as to further improve efficacy in the future. The ability to select patients and target visual field locations that are most likely helped by VRT enables these studies to investigate the effects precisely when and where they are expected to happen without diluting the datasets with samples for which no effects are expected. This is underscored by the GAS results in two ways. First it shows that GAS correlates with the extent of the recovered visual field, suggesting that visual rehabilitation effort may focus on partially recovering as many locations as possible. Yet, this hypothesis needs to be tested in future studies as well as the role of maximizing sensitivity at specific locations, which was not related to GAS in the current study. Secondly, GAS sets very different goals for peripheral and central vision loss. This means that the scores for patients with peripheral vision problems are not influenced by goals pertaining to central vision (e.g. reading) and vice versa. Many alternative measures do not make this distinction and therefore potentially assess goal achievement and quality of life in terms of factors that are irrelevant to a specific patient with a specific disease profile. Thus, we believe that the present findings represent a significant step forward in helping patients with chronic hemianopia as well as research into understanding visual cortex plasticity.

Subtle discrepancies between subjective visual field loss (i.e. detected by Humphrey perimetry) and objective visual field loss (i.e. detected by MRI) is also reported in Glaucoma (Murphy et al., 2016). While this study report reduced visual cortex activity prior to clinical visual field loss, our results show spared activity in the visual cortex while patients report to be blind. Despite the difference in clinical population (Glaucoma is an eye disease, whereas hemianopia is a consequence of brain damage), both studies show the importance to include the state of the brain on top of standard clinical testing to improve diagnosis and rehabilitation potential in patient groups with visual field loss.

### 4.2 Neural substrates and possible mechanisms of recovery

Interestingly, the cortical projections of the improved visual field locations were rather heterogenous, implicating early visual areas such as primary visual cortex in some patients, and various extrastriate regions in others. This inter-individual variability underscores the importance of using a neural marker of visual field coverage that is not restricted to one particular visual area (e.g. V1). We elected to perform neural perimetry based on responses across all visually driven cortical areas because it is challenging and often not possible to precisely delineate specific visual areas in patients with occipital lesions. The observation that many different cortical areas can contribute to the visual field recovery adds a second reason to not restrict the neural perimetry to particular visual areas. Indeed, it may be possible to further optimize the clinical impact of VRT by considering, on a case by case basis, the putative functions and stimulus preferences of affected brain areas.

The observed heterogeneity of neural correlates is also important in the light of previous hypotheses about the neural underpinnings of VRT (Das and Huxlin, 2010, Sabel et al., 2011, Hadid and Lepore, 2017, Das et al., 2014). As discussed in the introduction, there are several reasons for expecting mismatches between neural and behavioral perimetry. These depend on the nature and location of the neural injury, and different injury characteristics may afford different possible mechanisms supporting the visual field recovery induced by VRT. Two plausible hypotheses regarding these mechanisms are rooted in the ‘blindsight’ literature and involve the existence of spared islands of extant neural tissue that can exhibit neural activity in the absence of awareness (Baseler et al., 1999, Radoeva et al., 2008, Sahraie et al., 1997, Papanikolaou et al., 2019), or the re-initiation of latent visual pathways bypassing the lesioned visual areas (Das and Huxlin, 2010, Cowey, 2010). Based on the heterogenous projection maps observed in this work, it appears that both mechanisms have been at play in our cohort.

Based on the notion that recovery by VRT can be underpinned by different cortical locations at various stages along the visual processing hierarchy, we speculate that VRT invokes a generic mechanism that can operate across the entire visual cortex and possibly the entire brain. This mechanism would involve elevating subthreshold or distorted cortical activity to the levels required for visual awareness, which could in theory be achieved by both spared islands and bypassing pathways. It requires further work in patient groups with more homogenous lesion profiles to shed light on the underlying processes, though recent work from our group suggests that they might be related to attention mechanisms based on the observation that overall magnitude of the training effects (i.e. mean deviation change) is related to the strength of connectivity between visual cortex and precuneus (Halbertsma et al., 2020), which has previously been implicated in spatial attention modulation during saccade preparation and suppression. Indeed, the VRT taught patients to make successful covert attention shifts toward the experimental stimuli, which may well have boosted the attentional operations afforded by precuneus connections. We therefore speculate that VRT can lead to visual field recovery in a way that is conceptually related to how spatial attention brings specific visual objects into our awareness when preparing a saccade toward their location (Ritchie et al., 2012, Duhamel et al., 1992, Nakamura and Colby, 2002, Laamerad et al., 2020).

### 4.3 Methodological considerations

We used wide-field retinotopic mapping so as to enable roughly equal fields of view during behavioral and neural perimetry. Standard automated perimetry (i.e. under the SITA fast 30–2 protocol) assesses visual sensitivity at 76 perimetric grid locations covering the central 30 degrees of visual space, whereas fMRI typically assesses the central 15°or less. In addition, training effects are often observed peripherally beyond the central 15° (see for example Fig. 5A and 5B, and Supplemental Fig. S1), and there is no reason to expect that visual recovery is only possible for central vision. Indeed, just like the loss of central vision can have an enormous impact on a patient’s daily functioning, so can the loss of peripheral vision as evidenced by for instance the detrimental effects of Glaucoma. In similar vein, we did not want to exclude the possible involvement of regions with retinotopic representations that are biased toward the peripheral visual field. Thus, we believe that is important to not limit the neural perimetry to the central visual field in identifying possible training foci. That said, it should be noted that our current setup probably needs to be improved in terms of its applicability in clinical practice so as to avoid having the patient wear strong lenses and the requirement for precise calibration.

Another important feature of this work is that we did not attempt to delineate specific visual areas to then analyze the neural responses within those areas and convert them to area-specific neural perimetry maps. As mentioned above, we elected this approach because our hypothesis was not area-specific, and because accurate delineation of visual areas in lesioned brains can be very challenging. This last point in particular is not just relevant in the context of the present work but also in the wider context of possible clinical application, because it significantly simplifies the analytical procedures to the point that it should be possible to fully automate the production of neural perimetry maps, without the need for manual intervention and associated subjectivity. That said, it is important to note that the present neural perimetry maps combine responses across cortical areas with potentially different SNR levels as well as established differential receptive field characteristics and varying cortical magnification factors. These features have not been incorporated in the construction of the neural perimetry maps, which limits their quantitative interpretability. Indeed, it is for this reason—and also the reason that Humphrey perimetry uses very small light flashes whereas the fMRI experiment used large checkerboard stimuli—that we refrained from quantitatively comparing the degree of visual sensitivity between neural and behavioral perimetry, and it should be kept in mind that for the neural perimetry the sensitivities across grid locations are not directly comparable. In this context, it is also worth noting that based on cortical magnification, the likelihood of detecting neural responses to central visual stimulation is greater than for peripheral stimulation, which may seem at odds with the fact that we used a single detection threshold across the entire visual field. However, a single detection threshold is appropriate for the purposes of the present work, because visual sensitivity based on Humphrey perimetry also declines with eccentricity (and the results were robust to different detection thresholds as shown in Supplemental Fig. S2). Importantly, none of these considerations affect our conclusions, because they involve the simple presence or absence of neural responses whilst the training effects were established using Humphrey perimetry alone.

Another important consideration concerns the fact that the accuracy of both neural and Humphrey perimetry depends on the subject’s ability to maintain stable fixation. We therefore only included patients with the ability to maintain stable fixation during Humphrey perimetry according to standard clinical criteria for reliably perimetry. The patients could also maintain stable fixation during VRT: eye-tracking during training indicated fixations within the central 2°in 92% of all trials. The ability to maintain stable fixation during normal perimetry and VRT suggests that the patients could also maintain stable fixation during the neural perimetry, as was the case in previous work involving neural perimetry in patients with chronic hemianopia (Papanikolaou et al., 2014). However, whether that was indeed the case can only be demonstrated by simultaneous eye tracking, which was obstructed by the wide-field stimulus presentation setup. In the first group of patients, eye tracking was impossible because the projection screen was too close to the eye. For the second group, we adapted the projection screen slightly to enable simultaneous eye-tracking (by creating an opening in the projection screen in front of the eye without the lens), but only succeeded in acquiring valid datasets from two of the six patients included in the study. While these datasets indicated excellent central fixation stability (see Supplemental Fig. S4), we cannot rule out that some of the other patients did not maintain stable central fixation during the neural perimetry. If so, it is possible that in those patients the assignment of visual field locations to the different perimetric mismatch categories was inaccurate. However, it is unlikely that the ensuing noise in the estimates can explain the specificity with which the visual field location of training effects could be predicted. Rather, because patients had perimetric scotomas at varying visual field locations, it seems more likely that unstable fixation added noise and consequently underestimation of the effect sizes at the predicted mismatch locations (i.e. the blue bars in Fig. 4).

Finally, it should be noted that our study was designed to distinguish training-specific and non-specific visual field changes in a cross-over design (using training of the intact field as the control condition). In previous work, this allowed us to identify the specific effect of defect-training over and above of the non-specific effect of intact training (Elshout et al., 2016). For the purposes and experimental question of the present work, however, we consider the distinction between intact and defect training irrelevant, because the patient may profit from any improvement of the visual field. Hence, we used the full training effect at each location, (i.e., after training both the defect and the intact side of the visual field) to test the hypothesis that trainability occurs where the subject lacks awareness during standard perimetry where neural perimetry indicates residual visual processing.

## 5 Conclusions

In conclusion, we have shown that discrepancies between neural and standard behavioral perimetry define a subset of locations in the visual defect with neural reserve that are most susceptible to recovery by visual restitution training. Specifically, locations with neural reserve exhibited much stronger visual sensitivity gains than other locations. This result could be observed across two cohorts of patients whom performed different types of training paradigms. Back projections of the visual field locations onto visual cortex further indicated that the neural correlates of visual field recovery are heterogenous, implicating early visual areas in some patients and extrastriate regions in others. Finally, we observed that the number of visual field locations that recovered as a result of visual restitution training is related to overall satisfaction of the patient with the achieved result in relation to their own personal daily life activities and how that was impacted by the injury, thereby underscoring the clinical relevance of this work. Overall, our results represent an important advance in understanding neural plasticity in the face of chronic visual impairments due to stroke with important implications for the development of more targeted visual rehabilitation strategies.

## CRediT authorship contribution statement

**J.A. Elshout:** Conceptualization, Data curation, Formal analysis, Investigation, Methodology, Project administration, Visualization, Writing - original draft, Writing - review & editing. **D.P. Bergsma:** Conceptualization, Data curation, Investigation, Methodology, Supervision, Writing - original draft. **A.V. van den Berg:** Conceptualization, Formal analysis, Funding acquisition, Investigation, Methodology, Project administration, Resources, Software, Supervision, Writing - original draft, Writing - review & editing. **K.V. Haak:** Conceptualization, Formal analysis, Funding acquisition, Investigation, Methodology, Supervision, Visualization, Writing - original draft, Writing - review & editing.

## Declaration of Competing Interest

The authors declare that they have no known competing financial interests or personal relationships that could have appeared to influence the work reported in this paper.

## References

[b0005] Baseler H.A., Morland A.B., Wandell B.A. (1999). Topographic organization of human visual areas in the absence of input from primary cortex. J Neurosci..

[b0010] Baseler H.A., Gouws A., Haak K.V. (2011). Large-scale remapping of visual cortex is absent in adult humans with macular degeneration. Nat Neurosci..

[b0015] Bergsma D., Baars-Elsinga A., Sibbel J., Lubbers P., Visser-Meily A. (2014). Visual daily functioning of chronic stroke patients assessed by goal attainment scaling after visual restorative training: an explorative study. Top Stroke Rehabil..

[b0020] Bergsma D.P., van der Wildt G. (2010). Visual training of cerebral blindness patients gradually enlarges the visual field. Br J Ophthalmol..

[b0025] Carvalho J., Invernizzi A., Martins J., Jansonius N.M., Renken R.J., Cornelissen F.W. (2021). Visual Field Reconstruction Using fMRI-Based Techniques. Transl Vis Sci Technol..

[b0030] Chauhan B.C., Garway-Heath D.F., Goñi F.J. (2008). Practical recommendations for measuring rates of visual field change in glaucoma. Br J Ophthalmol..

[b0035] Cowey A. (2010). The blindsight saga. Exp Brain Res..

[b0040] Cowey A., Rolls E.T. (1974). Human cortical magnification factor and its relation to visual acuity. Exp Brain Res..

[b0045] Das A., Huxlin K.R. (2010). New approaches to visual rehabilitation for cortical blindness: outcomes and putative mechanisms. Neuroscientist..

[b0050] Das A., Tadin D., Huxlin K.R. (2014). Beyond blindsight: properties of visual relearning in cortically blind fields. J Neurosci..

[b0055] Duhamel J.R., Colby C.L., Goldberg M.E. (1992). The updating of the representation of visual space in parietal cortex by intended eye movements. Science..

[b0060] Dumoulin S.O., Wandell B.A. (2008). Population receptive field estimates in human visual cortex. Neuroimage..

[b0065] Elshout J.A., van Asten F., Hoyng C.B., Bergsma D.P., van den Berg A.V. (2016). Visual rehabilitation in chronic cerebral blindness: A randomized controlled crossover study. Front Neurol..

[b0070] Elshout J.A., van den Berg A.V., Haak K.V. (2018). Human V2A: A map of the peripheral visual hemifield with functional connections to scene-selective cortex. J Vis..

[b0075] Elshout J.A., Bergsma D.P., Sibbel J. (2018). Improvement in activities of daily living after visual training in patients with homonymous visual field defects using Goal Attainment Scaling. Restor Neurol Neurosci..

[b0080] Gall C., Franke G.H., Sabel B.A. (2010). Vision-related quality of life in first stroke patients with homonymous visual field defects. Health Qual Life Outcomes..

[b0085] Haak K.V., Cornelissen F.W., Morland A.B. (2012). Population receptive field dynamics in human visual cortex. PLoS One..

[b0090] Haak K.V., Langers D.R.M., Renken R., van Dijk P., Borgstein J., Cornelissen F.W. (2014). Abnormal visual field maps in human cortex: a mini-review and a case report. Cortex..

[b0095] Hadid V., Lepore F. (2017). From cortical blindness to conscious visual perception: theories on neuronal networks and visual training strategies. Front Syst Neurosci..

[b0100] Halbertsma H.N., Haak K.V., Cornelissen F.W. (2019). Stimulus- and Neural-Referred Visual Receptive Field Properties following Hemispherectomy: A Case Study Revisited. Neural Plast..

[bib231] Halbertsma H.N., Elshout J.A., Bergsma D.P. (2020). Functional Connectivity of the Precuneus Reflects Effectiveness of Visual Restitution Training in Chronic Hemianopia. NeuroImage Clinical.

[b0105] Huxlin K.R., Martin T., Kelly K. (2009). Perceptual relearning of complex visual motion after V1 damage in humans. J Neurosci..

[b0110] Kasten E., Wüst S., Behrens-Baumann W., Sabel B.A. (1998). Computer-based training for the treatment of partial blindness. Nat Med..

[b0115] Khan A.Z., Song J.-H., McPeek R.M. (2011). The eye dominates in guiding attention during simultaneous eye and hand movements. J Vis..

[b0120] Laamerad P., Guitton D., Pack C.C. (2020). Eye movements shape visual learning. Proc Natl Acad Sci USA.

[b0125] Marshall R.S., Chmayssani M., O’Brien K.A., Handy C., Greenstein V.C. (2010). Visual field expansion after visual restoration therapy. Clin Rehabil..

[b0130] Miranda ÂSC, Martins Rosa A de F, Patrício Dias MJ, et al. Optical properties influence visual cortical functional resolution after cataract surgery and both dissociate from subjectively perceived quality of vision. Invest Ophthalmol Vis Sci. 2018;59(2):986-994. 10.1167/iovs.17-22321. 10.1167/iovs.17-22321 29450542

[b0135] Murphy M.C., Conner I.P., Teng C.Y. (2016). Retinal structures and visual cortex activity are impaired prior to clinical vision loss in glaucoma. Sci Rep..

[b0140] Nakamura K., Colby C.L. (2002). Updating of the visual representation in monkey striate and extrastriate cortex during saccades. Proc Natl Acad Sci USA.

[b0145] Papageorgiou E., Hardiess G., Schaeffel F. (2007). Assessment of vision-related quality of life in patients with homonymous visual field defects. Graefes Arch Clin Exp Ophthalmol..

[b0150] Papanikolaou A., Keliris G.A., Papageorgiou T.D. (2014). Population receptive field analysis of the primary visual cortex complements perimetry in patients with homonymous visual field defects. Proc Natl Acad Sci USA.

[b0155] Papanikolaou A., Keliris G.A., Papageorgiou T.D., Schiefer U., Logothetis N.K., Smirnakis S.M. (2019). Organization of area hV5/MT+ in subjects with homonymous visual field defects. Neuroimage..

[b0160] Plow E.B., Obretenova S.N., Fregni F., Pascual-Leone A., Merabet L.B. (2012). Comparison of visual field training for hemianopia with active versus sham transcranial direct cortical stimulation. Neurorehabil Neural Repair..

[b0165] Radoeva P.D., Prasad S., Brainard D.H., Aguirre G.K. (2008). Neural activity within area V1 reflects unconscious visual performance in a case of blindsight. J Cogn Neurosci..

[b0170] Ritchie K.L., Hunt A.R., Sahraie A. (2012). Trans-saccadic priming in hemianopia: sighted-field sensitivity is boosted by a blind-field prime. Neuropsychologia..

[b0180] Sabel B.A., Henrich-Noack P., Fedorov A., Gall C. (2011). Vision restoration after brain and retina damage: the “residual vision activation theory”. Prog Brain Res..

[b0185] Sahraie A., Weiskrantz L., Barbur J.L., Simmons A., Williams S.C., Brammer M.J. (1997). Pattern of neuronal activity associated with conscious and unconscious processing of visual signals. Proc Natl Acad Sci USA.

[b0190] Sahraie A., Trevethan C.T., MacLeod M.J., Murray A.D., Olson J.A., Weiskrantz L. (2006). Increased sensitivity after repeated stimulation of residual spatial channels in blindsight. Proc Natl Acad Sci USA.

[b0195] Silson E.H., Aleman T.S., Willett A. (2018). Comparing Clinical Perimetry and Population Receptive Field Measures in Patients with Choroideremia. Invest Ophthalmol Vis Sci..

[b0200] Silvanto J., Cowey A., Lavie N., Walsh V. (2007). Making the blindsighted see. Neuropsychologia..

[b0205] Sims J.R., Chen A.M., Sun Z. (2020). Role of structural, metabolic, and functional MRI in monitoring visual system impairment and recovery. J Magn Reson Imaging..

[b0210] Turner-Stokes L., Ashford S. (2007). Serial injection of botulinum toxin for muscle imbalance due to regional spasticity in the upper limb. Disabil Rehabil..

[b0215] van den Berg A.V. (1996). Judgements of heading. Vision Res..

[b0220] Weiskrantz L., Warrington E.K., Sanders M.D., Marshall J. (1974). Visual capacity in the hemianopic field following a restricted occipital ablation. Brain..

[b0225] Zhang X., Kedar S., Lynn M.J., Newman N.J., Biousse V. (2006). Natural history of homonymous hemianopia. Neurology..

[b0230] Zihl J. Rehabilitation of visual disorders after brain injury. 2010. 10.1016/j.cortex.2012.04.008 22622434

