# Supplementary material for: Functional MRI of visual cortex predicts training-induced recovery in stroke patients with homonymous visual field defects
Source: Neuroimage Clin. 2021 May 21;31:102703. doi: 10.1016/j.nicl.2021.102703 (PMC8173295; doi:10.1016/j.nicl.2021.102703)
Supplement: Supplementary data 1 [file mmc1.pdf]

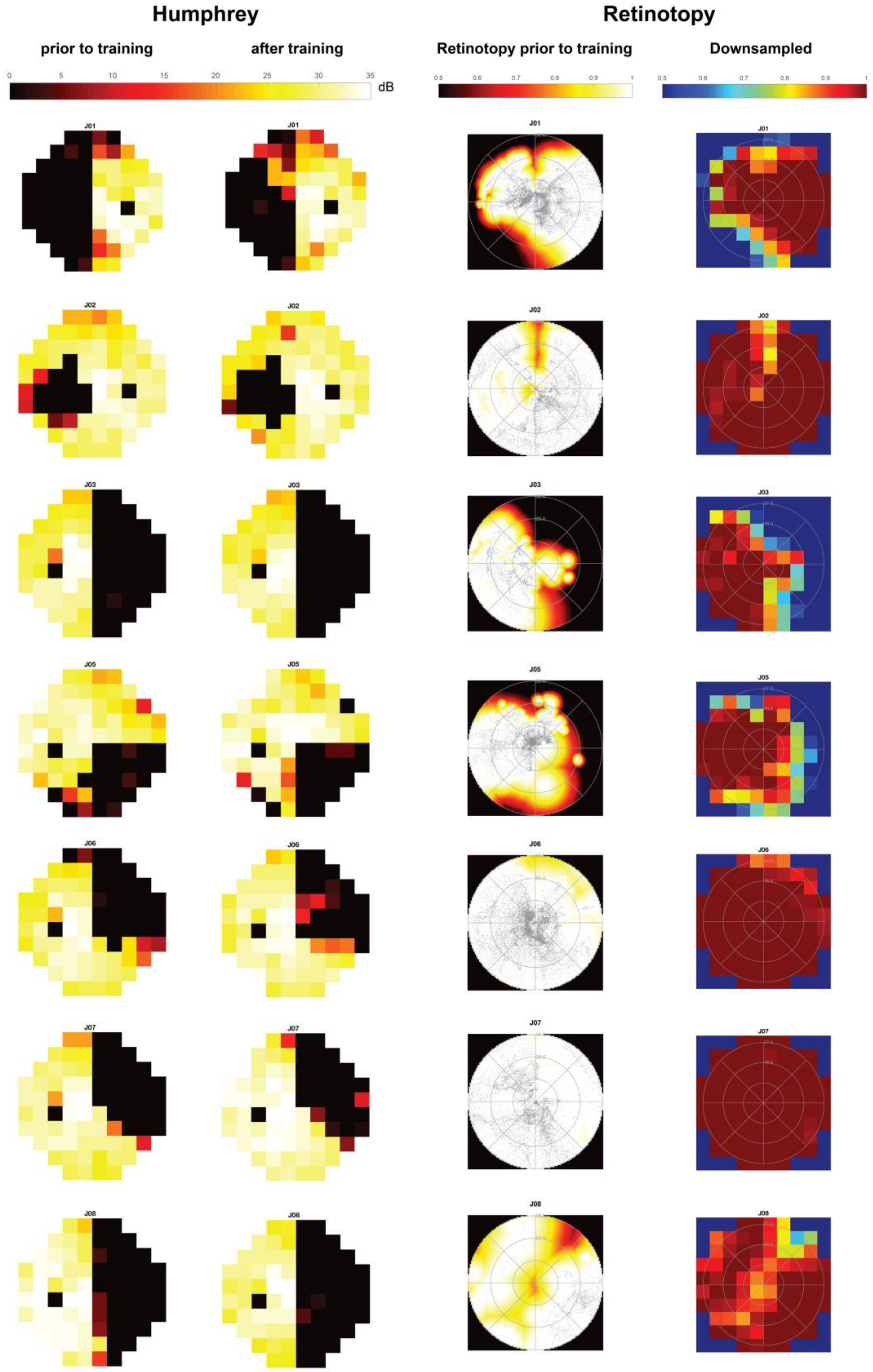

Figure S1: Humprey and retinotopy. The first 2 columns represent the Humphrey data prior to and after training. The third column is the retinotopic mapping data and the last column represent the downsampled retinotopic data to match the Humphrey resolution. The other patients are shown on the next pages.

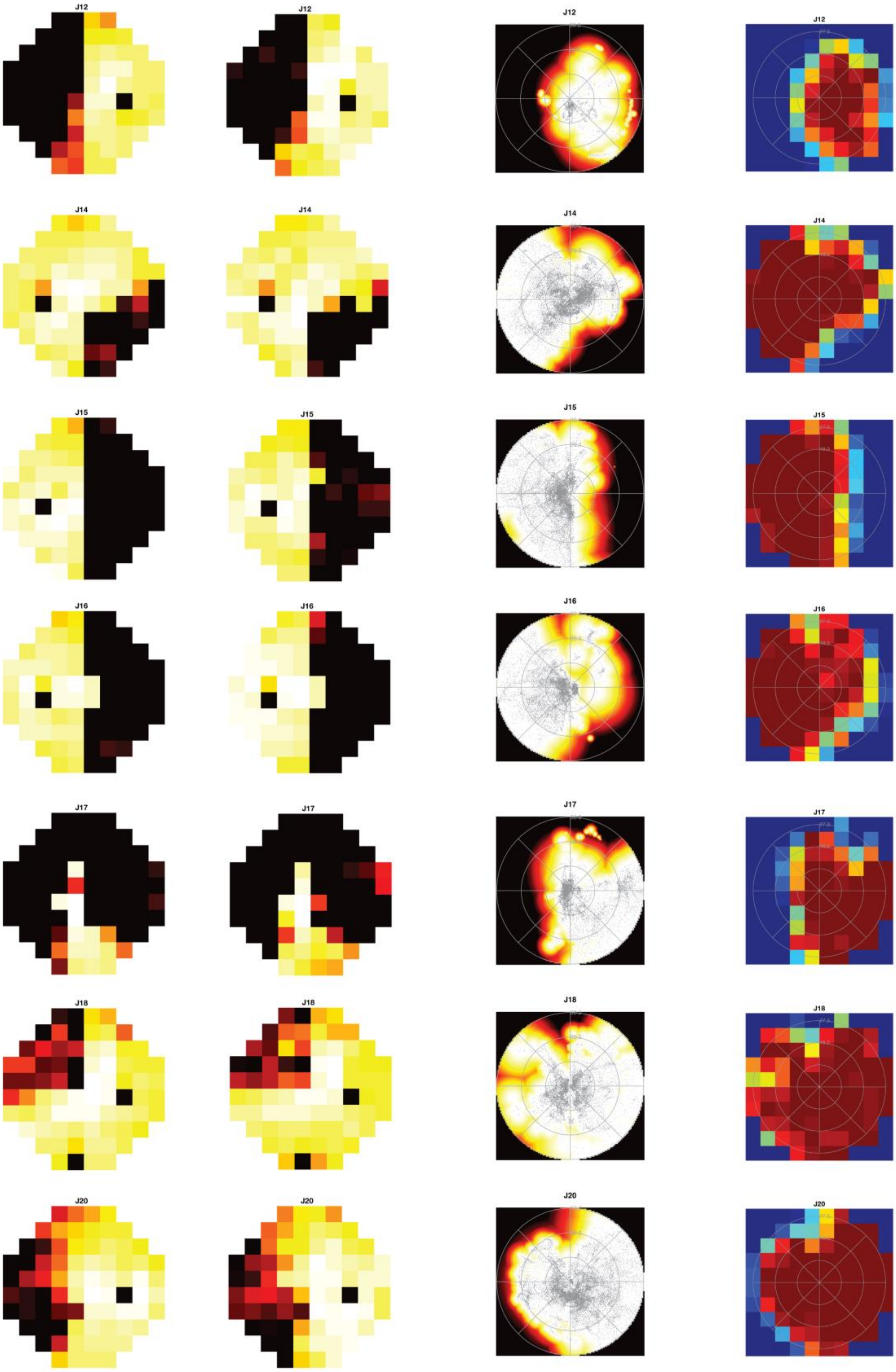

Figure S1: J12-J20

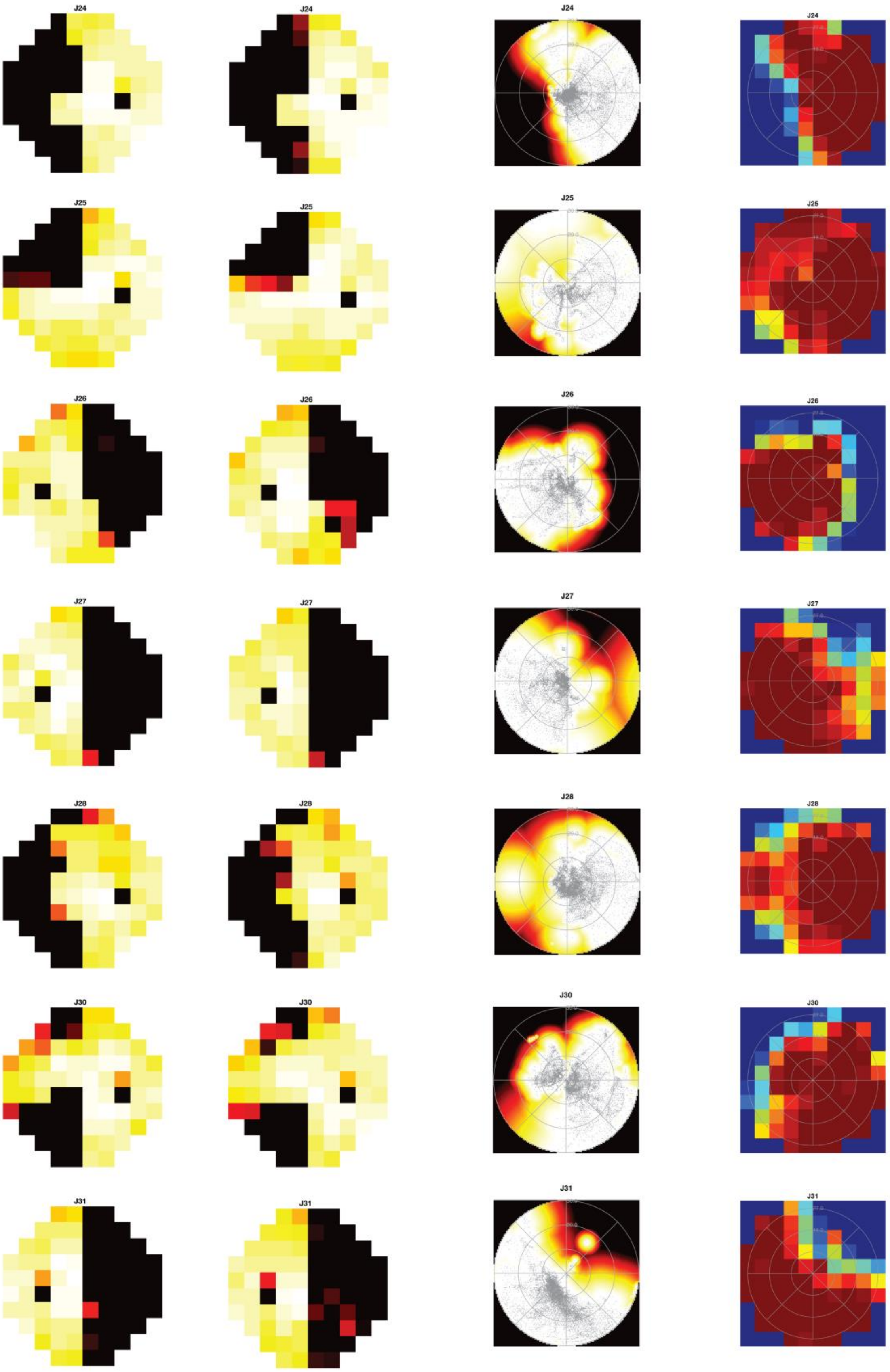

Figure S1: J24-J31

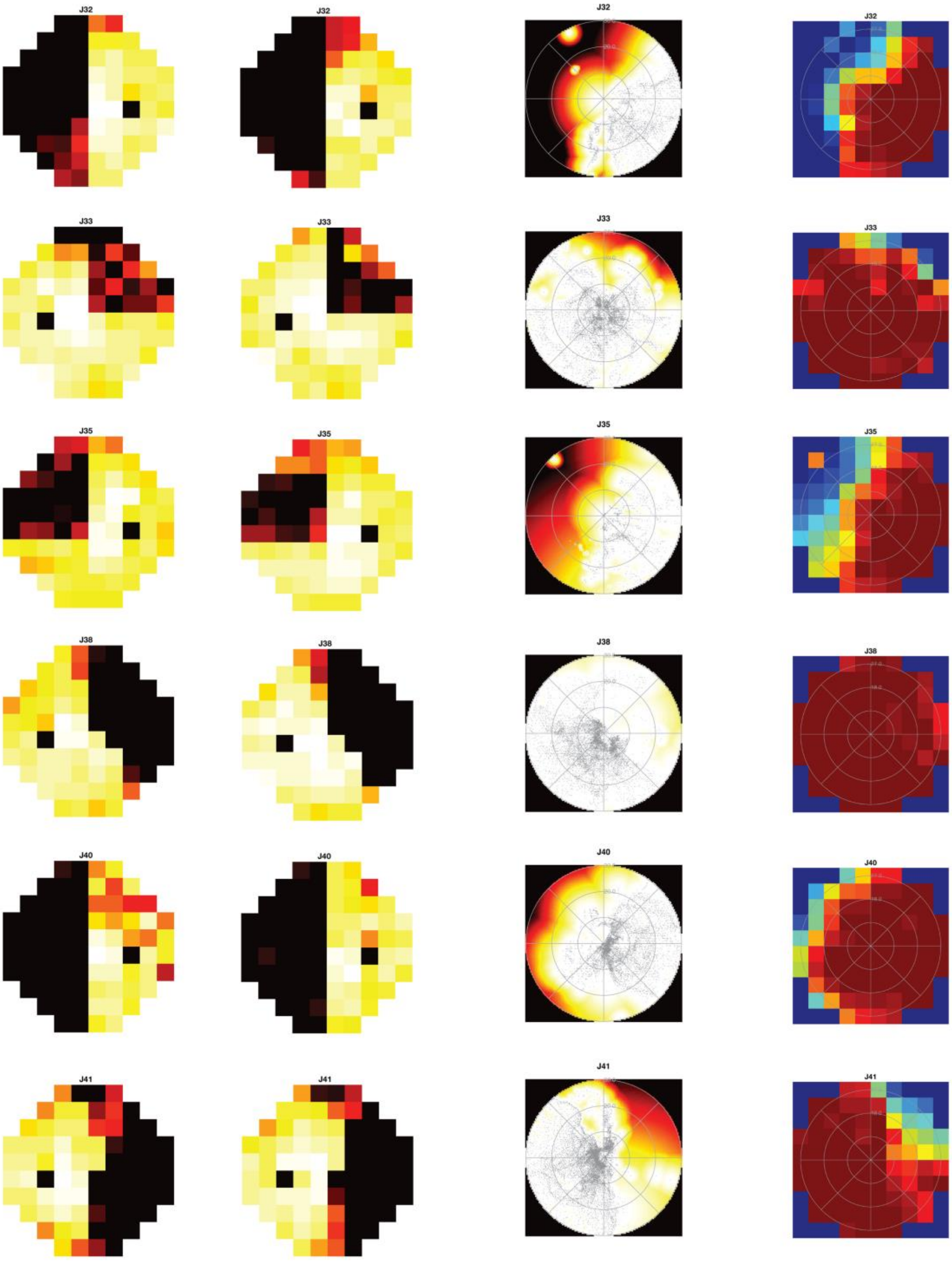

Figure S1: J32-J41

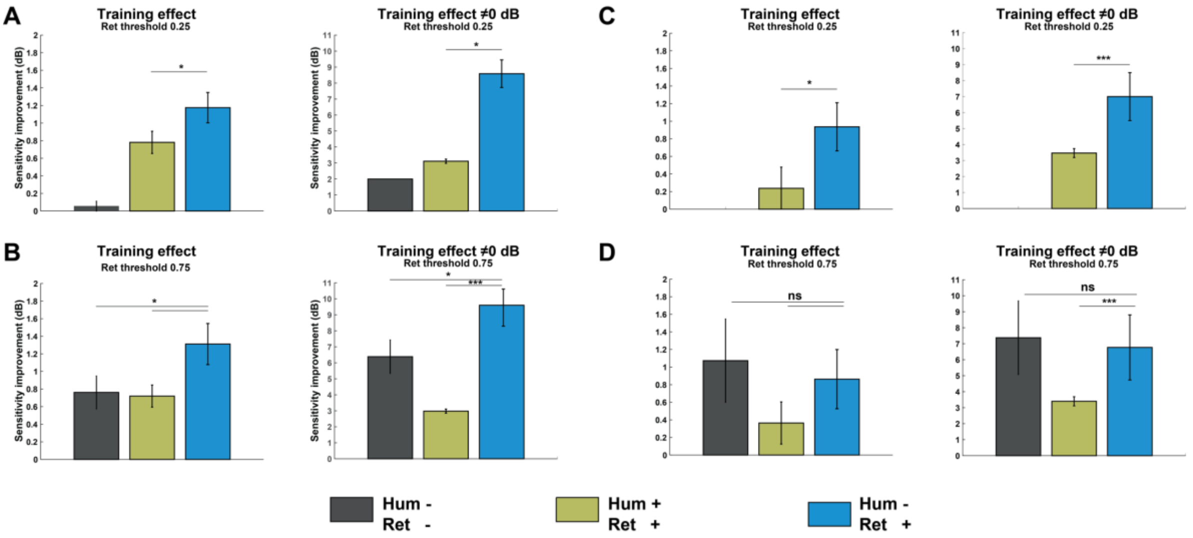

Figure S2: Training effect based on different thresholds of the retinotopic coverage maps. A) Threshold at 0.25 training group A (n=21). B) Threshold at 0.75 training group A (n=21). This analysis reveals that the training effect in the Hum-/Ret + category (blue bars) is highest irrespective of the chosen thresholds of the retinotopic map. C) Threshold at 0.25 training group B (n=6). D) Threshold at 0.75 training group B (n=6). The same analysis can be partly replicated in the smaller sample of training group B.

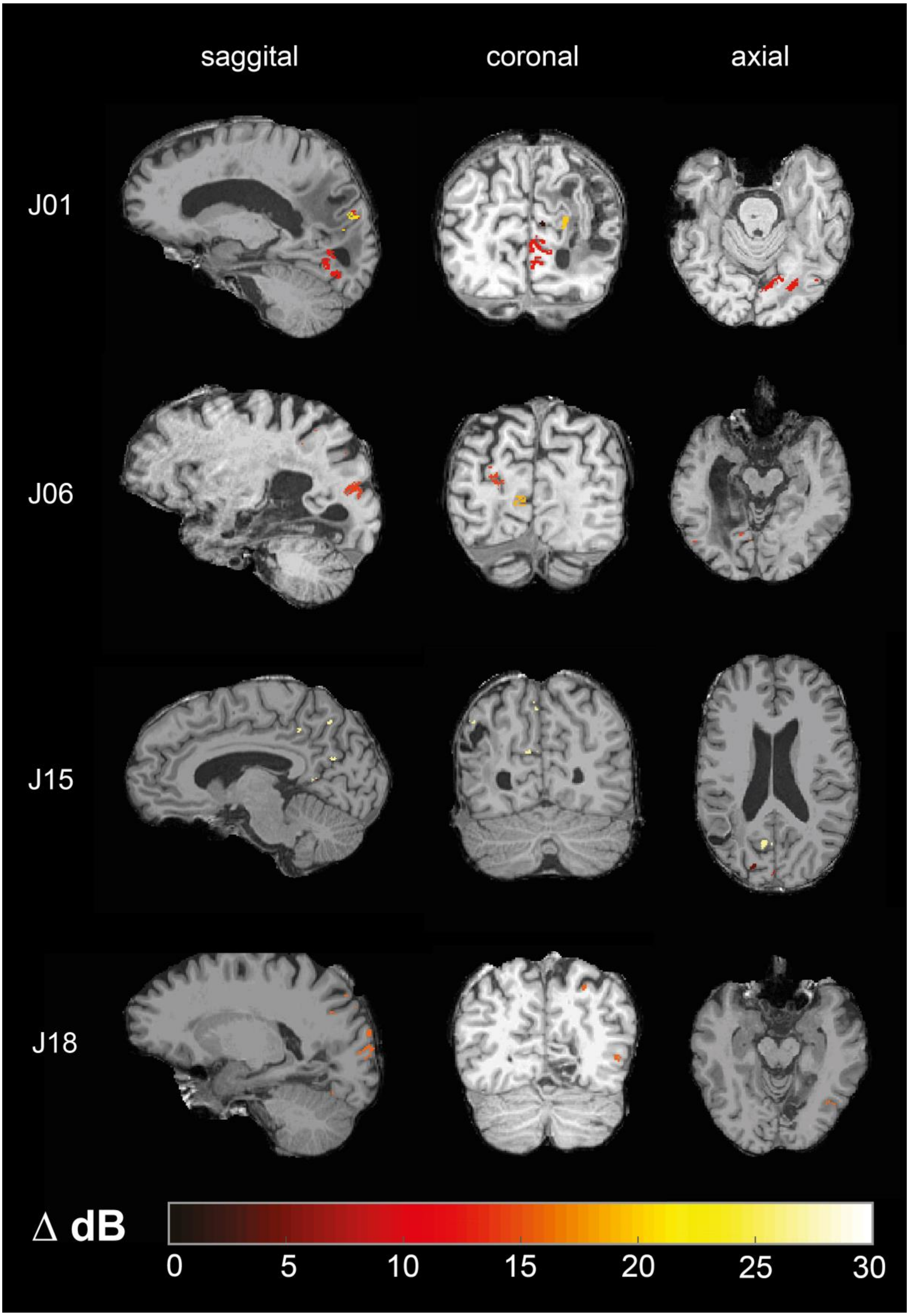

Figure S3: Locations with training effect (sensitivity gain based on Humphrey perimetry) of patient J01, J06, J15 and J18.

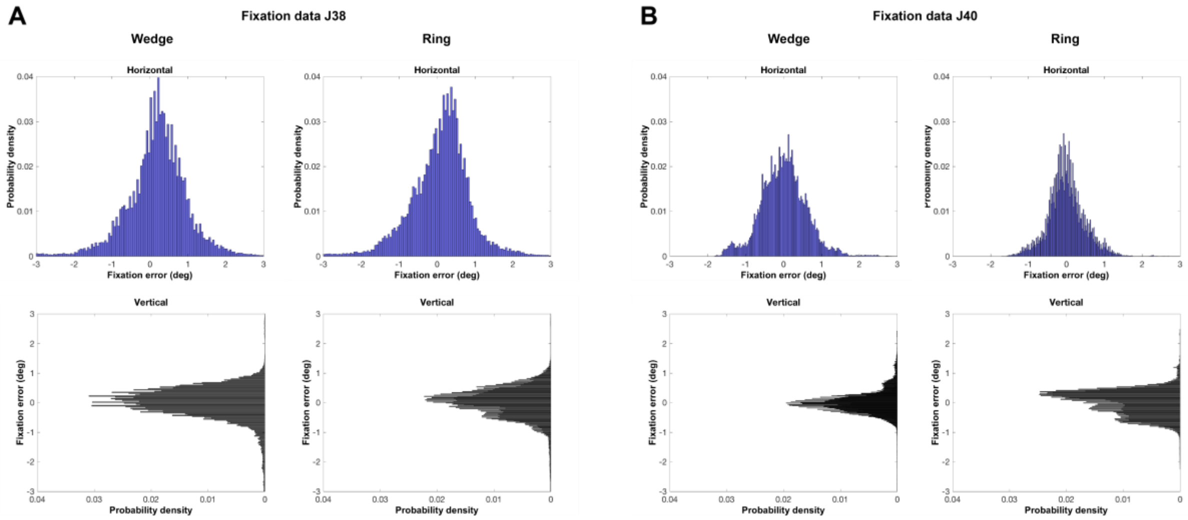

Figure S4: Fixation data during wide field retinotopic mapping of 2 patients. A) Fixation data of J38. B) Fixation data of J40.
Fixation of both patients was >96% of the time within 2deg of the fixation point. Upper left panels represent the distribution of horizontal fixation errors across all wedge stimuli (4 repetitions). Upper right panels represent the distribution of horizontal fixation errors across all ring stimuli (4 repetitions). The lower left panels represent the distribution of vertical fixation errors.

Table S1: Humphrey characteristics

	Pre measurement			Post measurement		
Subject	Fixation errors (%)	False Positive (%)	False Negative (%)	Fixation errors (%)	False Positive (%)	False Negative (%)
J01	7.1	0	0	0	0	0
J02	20	0	0	0	0	0
J03	0	0	0	7.6	0	0
J05	7.1	0	0	0	4	0
J06	7.6	1	0	0	2	12
J07	0	0	0	0	0	0
J08	0	0	0	0	0	0
J41	0	0	33	0	1	11
J12	0	1	0	0	5	0
J14	0	3	0	1	5	0
J15	0	2	0	15.3	9	12
J16	0	0	0	0	4	0
J17	6.2	2	0	5.8	3	14
J18	0	0	0	0	1	0
J20	13.3	3	0	13.3	5	0
J24	0	0	0	7.1	1	0
J25	8.3	6	0	8.3	0	12
J26	7.1	2	0	0	2	0
J27	0	0	0	0	1	0
J28	7.1	1	0	6.6	0	0
J30	0	6	37	0	3	0
J31	0	0	0	7.6	6	0
J32	0	0	12	0	0	11
J33	7.6	2	0	0	0	0
J35	14.2	8	0	0	1	0
J38	7.1	3	11	0	5	0
J40	0	4	0	0	3	0
mean (SE)	4.1 (1)	1.6 (0.4)	3.4 (1.9)	2.7 (0.9)	2.3 (0.5)	2.7 (1)
